# Microwave-Assisted Hydro-Distillation of Essential Oil from Rosemary: Comparison with Traditional Distillation

**Published:** 2018

**Authors:** Sara Moradi, Alireza Fazlali, Hamid Hamedi

**Affiliations:** 1. Department of Chemical Engineering, Arak University, Arak, Iran; 2. Department of Chemical Engineering, University of Science and Technology, Tehran, Iran

**Keywords:** Essential Oil, Hydro distillation, Microwave distillation, Rosemary

## Abstract

**Background:**

Hydro-distillation (HD) method is a traditional technique which is used in most industrial companies. Microwave-assisted Hydro-distillation (MAHD) is an advanced HD technique utilizing a microwave oven in the extraction process.

**Methods:**

In this research, MAHD of essential oils from the aerial parts (leaves) of rosemary (*Rosmarinus officinalis L.*) was studied and the results were compared with those of the conventional HD in terms of extraction time, extraction efficiency, chemical composition, quality of the essential oils and cost of the operation.

**Results:**

Microwave hydro-distillation was superior in terms of saving energy and extraction time (30 *min*, compared to 90 *min* in HD). Chromatography was used for quantity analysis of the essential oils composition. Quality of essential oil improved in MAHD method due to an increase of 17% in oxygenated compounds.

**Conclusion:**

Consequently, microwave hydro-distillation can be used as a substitute of traditional hydro-distillation.

## Introduction

*Rosmarinus officinalis L.*, commonly known as rosemary, is a woody, perennial herb with fragrant, evergreen, needle-like leaves native to the Mediterranean region. Rosemary’s antioxidant properties are still used to extend the shelf life of prepared foods ^1–3^ . Rosemary is also known medicinally for its powerful antioxidant activity and antibacterial properties ^4^ and as a chemopreventive agent ^5^ . Today, essential oil of rosemary is widely used in the cosmetic industry producing various bathing essences, hair lotions and shampoos ^6^ .

Traditional methods used for extraction of essential oil were Hydro-distillation (HD) or steam distillation. However, the loss of some components and the degradation of some unsaturated compounds by thermal effects ^7,8^ or by hydrolysis can take place by these conventional extraction techniques. These disadvantages have changed the approach of the recent research and stimulated the intensification, optimization and improvement of existing and novel “green” extraction techniques ^9,10^ . Some of these methods are: Ultrasound-Assisted Extraction, Microwave-Assisted Extraction and CO _2_ supercritical extraction. The supercritical fluid extraction of rosemary with CO _2_ has been the object of many researches ^11^ and has become a valid alternative to the more conventional extraction procedures, mainly because the dissolving power of the extracting medium can be adjusted by regulating pressure and temperature conditions. However, the high cost of producing specific products has limited its use. In certain cases, the extractive power of supercritical CO _2_ is insufficient under conventional conditions. Microwave-assisted solvent extraction appeared to be particularly attractive for isolation of essential oil ^12,13^ . Microwave-assisted Hydro-distillation (MAHD) combines rapid heating in the microwave field with the traditional solvent extraction. This significantly enables saving of time, so the extraction can be completed in meter of minutes ^14,15^ . In an attempt to take advantage of microwave heating with the conventional HD, MAHD was developed and used for the extraction of essential oils from *Xylopia aromatica* (Lamarck) and *Lippia alba* (Mill) ^16,17^ .

Tigrine-Kordjani *et al* ^18^ developed a Microwave Assisted Distillation (MAD) with free solvent for laboratory scale applications in the extraction of essential oils from different kinds of aromatic plant. Sui *et al* ^19^ have worked on an efficient Microwave Pretreatment (MP) method to maintain quality of postharvest rosemary leaves and observed that MP could be a good method for extracting essential oil and maintaining quality in rosemary and other aromatic herbs. Lucchesi *et al* ^20^ had a study on comparing solvent-free microwave extraction of essential oil from aromatic herbs with conventional hydro-distillation. They found that the SFME method yields an essential oil with higher amounts of more valuable oxygenated compounds, and allows substantial savings of costs, in terms of time, energy and plant material. SFME is a green technology and appears as a good alternative for the extraction of essential oils from aromatic plants.

In this paper, the essential oil from rosemary obtained by microwave hydro-distillation has been compared with those obtained by conventional hydrodistillation. Then, the quality and quantity of essential oil, cost, energy consumption and safety environmental consideration of two methods were studied. Also, different parameters on essential oil extraction quantity and quality were checked.The aim of this research was finding an optimum method for extraction of essential oil.

## Materials and Methods

### Material

Leaves of the cultivated plants of rosemary (*Rosmarinus officinalisL.*) were collected from Arak and Isfahan Universities. The leaves were dried in shade for two weeks.

### Hydro-Distillation (HD) apparatus and procedure

100 *gr* of rosemary leaves were submitted to hydro-distillation with a clevenger-type apparatus with a maximum delivered power of 1000 *W*. The essential oil was extracted with 300 *ml* of water in a 2 *L* flask for 90 *min* (until no more essential oil was obtained). Then, the essential oil was collected and stored at laboratory condition.

### Microwave-Assisted Hydro-Distillation (MAHD) apparatus and procedure

**Figure 1.**
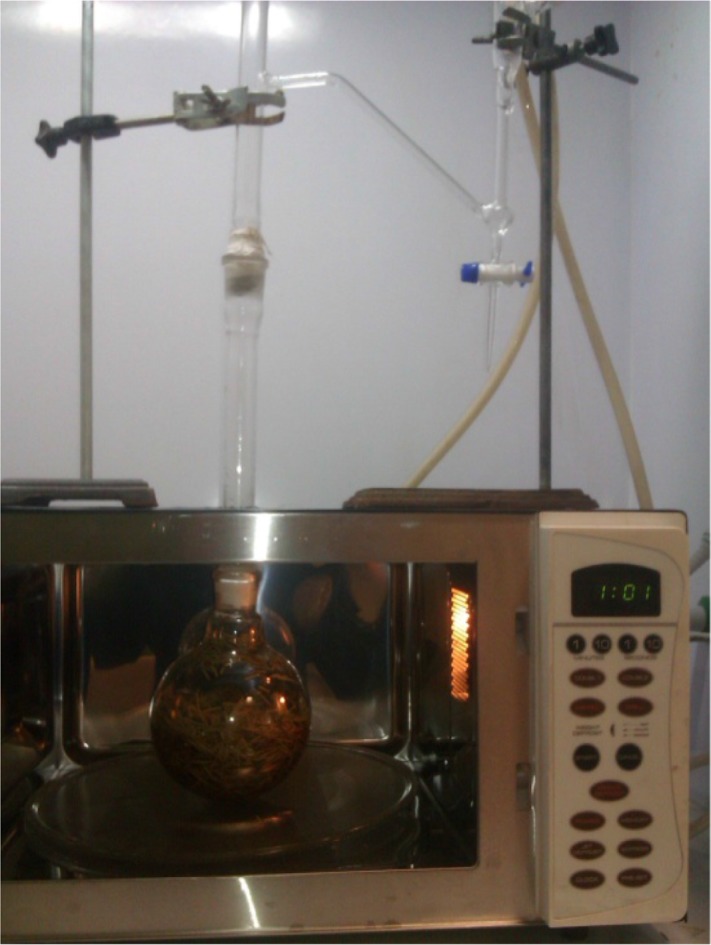
MAHD apparatus used in this study.

Microwave hydro-distillation has been performed using the Tecnokit Chen (Italy, Tek-2611) microwave oven (Figure 1). It is a 2450 *MHz* multimode microwave with a maximum delivered power of 900 *W*. In a typical MAHD procedure performed at atmospheric pressure, 100 *gr* of rosemary were heated for 30 *min* with addition of 300 *ml* water. This period was sufficient to extract all the essential oils from the sample. Each extraction was performed at least three times.

### Gas chromatography

The essential oils obtained at different conditions were analyzed by gas chromatography. The capillary column used for the analysis was HP-5MS (30 *m*×0.25 *mm*×0.25 *μm*) with a 5% phenyl methyl siloxane stationary phase. The GC analysis was performed with the following conditions: flow rate, 0.4 *ml/min*; FID temperature, 275°*C*; make-up gas type, He with a make-up flow rate of 45 *ml/min*. Identification of the components of the essential oils were done by comparing data with those of literature ^12, 21, 22^ which is based on comparison of retention times of compounds with those of available standards and with library matching of their spectra. Most of the components except those in table 1 were identified based on the information reported by Adams ^22^ . The identification of individual components is not always possible using MS data alone; the use of chromatographic information, such as retention index, facilitates more reliable peak assignment of components in complex mixtures ^23^ .

**Table 1.** Concentrations of the essential oil of Rosemary compounds obtained by different methods

**No.**	**Component**	**Ret. Time**	**Ret. Index**		**Concentration (*mg/ml*)**			

		***min***	Lit.*	Exp.**	**HD**	**MAHD (40%)**	**MAHD (60%)**	**MAHD(40%)+NaCl**
**1**	Tricyclene	9.84	926	922	0.07	0.05	-	-
**2**	a-Thujene	13.39	931	926	0.31	0.25	0.21	0.28
**3**	a-Pinene	13.61	939	930	24.24	20.02	17.95	22.74
**4**	Camphene	13.96	953	950	7.51	6.4	5.35	6.95
**5**	β-pinene	14.02	980	976	4.27	4.79	4.32	5.59
**6**	1-octen-3-ol	14.28	978	975	0.34	0.49	0.41	0.5
**7**	β-myrcene	14.36	991	985	2.48	2.3	2.14	2.37
**8**	α-phellandrene	14.52	1005	1000	0.33	0.3	0.26	0.3
**9**	3-carene^18^	14.97	1011	1008	0.05	0.15	0.13	-
**10**	α-terpinene	15.20	1018	1015	1.64	1.59	1.52	1.38
**11**	P-cymene	15.44	1026	1022	3.74	3.5	3.46	3.52
**12**	1,8-cineole^18^	15.52	1032	1030	8.79	9.35	8.84	9.34
**13**	Cis-Ocimene	15.92	1040	1036	0.09	0.08	-	0.09
**14**	β-Ocimene	16.13	1050	1045	0.05	0.06	-	-
**15**	γ-terpinene	16.45	1062	1060	2.36	3.49	3.33	3.28
**16**	cis-Sabinene hydrate^18^	16.72	1067	1065	0.69	0.74	0.66	0.74
**17**	Terpinolene	17.15	1088	1085	0.32	0.38	0.3	0.22
**18**	Linalool	17.73	1098	1090	10.98	11.99	12.19	12.13
**19**	Fenchol	17.80	1115	1110	0.06	0.07	-	-
**20**	Campholaldehyde^18^	17.96	1124	1120	2.13	2.35	2.39	2.23
**21**	Camphor	18.20	1143	1140	7.56	8.75	8.86	8.06
**22**	Isopulegol	18.28	1145	1147	1.49	1.71	1.8	1.62
**23**	Pinocamphone/isopinocamphone	18.46	1160	1162	1.83	2.15	2.24	1.91
**24**	Pinocarvone/trans-pinocarvone	18.60	1162	1164	1.25	1.48	1.56	1.32
**25**	Borneol	18.81	1165	1167	11.28	11.53	15.38	10.39
**26**	4-terpineol^16^	19.25	1177	1175	0.19	0.21	0.23	0.2
**27**	p-cymene-8-ol	19.41	1183	1180	0.77	0.95	1.01	0.82
**28**	α-terpineol	19.55	1189	1185	1.17	1.39	1.43	1.25
**29**	Myrtenol	19.72	1194	1195	0.04	0.05	0.13	-
**30**	Verbenone	20.16	1204	1200	1.49	1.66	1.72	1.33
**31**	Citronellol	20.60	1228	1225	0.04	0.05	-	-
**32**	Bornyl acetate	20.99	1285	1280	0.33	0.29	0.42	0.27
**33**	Carvacrol	21.40	1298	1295	0.27	0.05	-	-
**34**	Methyl eugenol	21.54	1401	1400	0.13	0.1	-	0.08
**35**	β-caryophyllene	22.93	1418	1415	0.3	0.5	0.59	0.49
**36**	α-humulene	23.44	1453	1450	0.06	0.08	-	0.11
**37**	Methyl jasmonate	25.57	1647	1650	0.1	0.13	0.19	0.14
**38**	Others	26.52			0.35	0.35	0.51	0.29
	**Total**				**98.6**	**99.21**	**99.23**	**99.61**
	**Monoterpene hydrocarbon**				**47**	**42.84**	**38.67**	**46.39**
	**Oxygenated compounds**				**50.88**	**55.44**	**59.46**	**52.33**
	**Sesquiterpenes**				**0.36**	**0.58**	**0.59**	**0.6**

NI : Not identified,

*: Literature,

**: Experimental

## Results

### Effect of extraction methods on the essential oil efficiency

**Figure 2.**
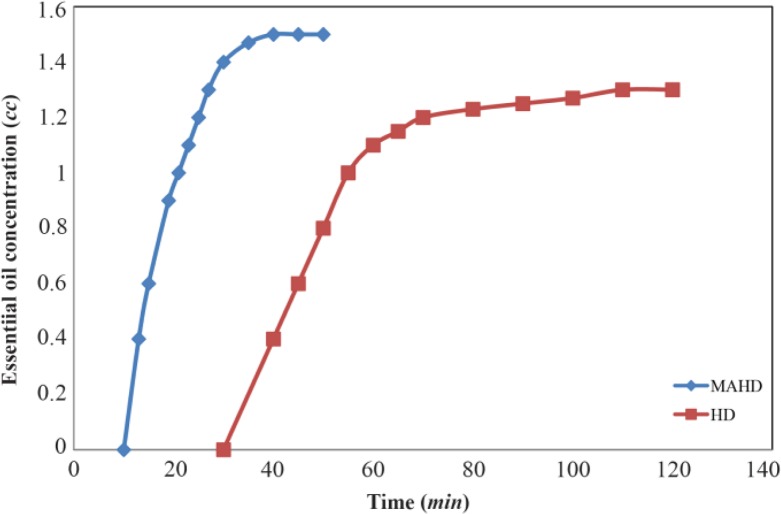
Extraction yield as a function of time for the hydro-distillation. (HD) and microwave-assisted hydro-distillation (MAHD) of essential oils from thyme aerial parts.

All essential oils extracted from the aerial parts of rosemary by both extraction methods produced a clear, yellow liquid essential oil. Efficiency was expressed in terms of the volume of the oil collected in *ml/gm* of dry plant material. Efficiency of essential oil extraction versus time of extraction for both methods is shown in figure 2. Extraction with MAHD started at much earlier time than that with HD (10 *min vs*. 30 *min*, respectively). This is due to the more efficient heat flow involved in microwaves. Microwave can heat the entire sample almost simultaneously and at a higher rate ^24^ . Extraction of essential oils was achieved within 30 *min* of operation with MAHD. In comparison, HD requires a time period of at least 90 *min* to be completed.

For both HD and MAHD, the extraction starts at the boiling point of water (100°*C*, at atmospheric pressure). The essential oil extracted by MAHD and HD was about 1.5 *cc* and 1.3 *cc*, respectively.

These results confirm results from the literature, which indicate that the use of microwaves allows extractions to be accelerated ^25,26^ .

### Effect of different microwave power on efficiency

An appropriate microwave irradiation power is important to ensure that essential oil is extracted quickly. However, the power should not be too high otherwise loss of volatile compounds would result ^13^ . Different microwave irradiation power, 180, 360 and 540 *W* (20, 40 and 60% of maximum oven power respectively), were examined for MAHD essential oils extraction. Then, the total extraction time in relation with the microwave irradiation power was studied. Higher and lower power of microwave due to high intensity of heating to the mixture and possibility of degradation of thermolabile components and low speed of essential oil extraction was left out, respectively. So the microwave power of 360 *W* for 100 *gr* of plant material was chosen as the best power density. The total amount of extracted essential oil in this case was about 1.5 *ml*.

### Addition of salt

In the next experiment, 30 *g* salt (NaCl) was solved into 300 *ml* water and added to rosemary. NaCl salt increases the boiling point of water. So salt was added to the mixture to check that it will extract higher amounts of essential oil or not. Addition of salt led to a faster extraction operation but the extraction efficiency didn’t improve as expected. Salt accelerates transfer phenomenon but may cause thermal and hydrolysis reactions and can degrade thermolabile components of essential oil. So this option was left out.

### Effect of extraction methods on the essential oil composition

**Figure 3.**
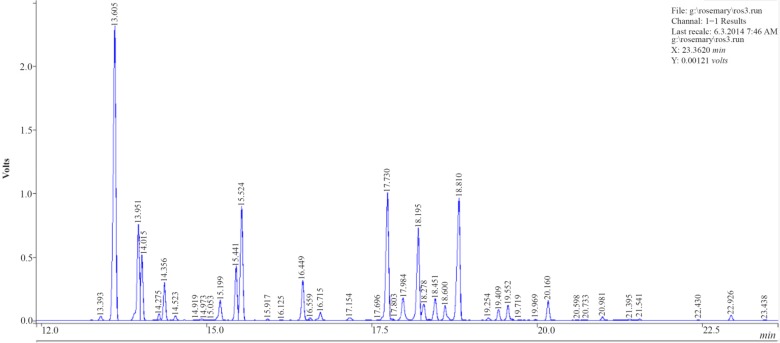
Chromatography of Rosemary essential oil by HD.

**Figure 4.**
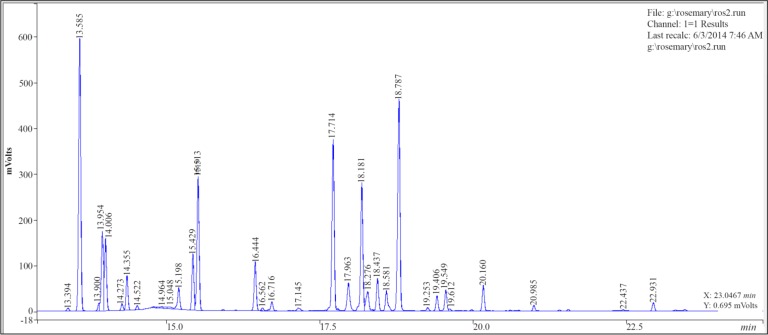
Chromatography of Rosemary essential oil by MAHD.

The total chromatography of the rosemary essential oil by HD and MAHD are given in figures 3 and 4.

The composition of the essential oil of rosemary obtained by both methods is given in table 1. The compositions of the essential oils obtained by both methods were found to be almost the same qualitatively, where-as some quantitative differences were observed.

The main components of the rosemary essential oil were obtained a-Pinene, Camphene, 1,8-Cineole, Linalool, Camphor and Borneol. It was also found that the essential oil mainly composed of oxygenated compounds 51.35–59.74% while monoterpene hydrocarbons and sesquiterpenes constituted 38.86–47.43% and 0.36–0.6% of it, respectively. The main component of oxygenated compounds and monoterpene hydrocarbons detected were Borneol (10.39–15.38 *mg/ml*) and a-Pinene (17.95–24.24 *mg/ml*) in the case of MAHD.

**Figure 5.**
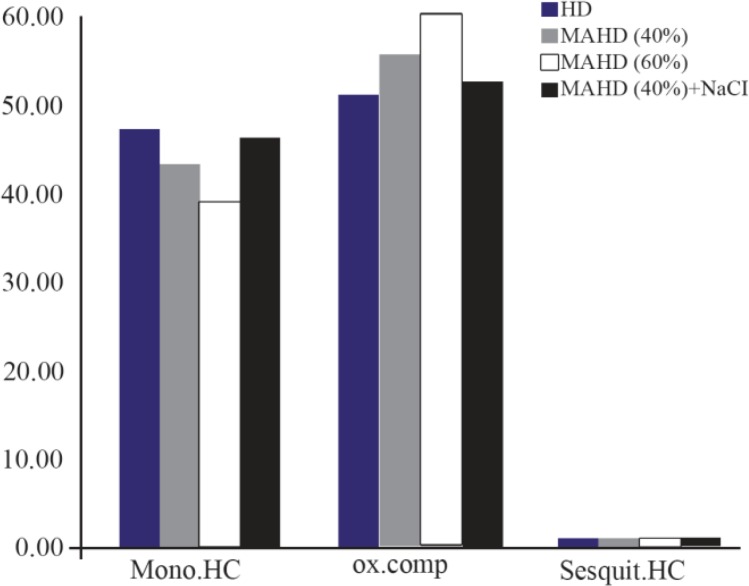
Comparison between chemical compositions of rosemary extracts isolated through the different processes. HC=hydrocarbons, OX=oxygenated.

The general chemical profile of the samples investigated is given in figure 5; the profiles are expressed as average relative quantities and refer to three chemical classes: monoterpene hydrocarbons, sesquiterpenes and oxygenated compounds. Concentration percent of these chemicals are given in table 2.

**Table 2.** Percent of chemical components of rosemary extracts by different methods

	**HD**	**MAHD (40%)**	**MAHD (60%)**	**MAHD (40%)+NaCl**
**Monoterpene hydrocarbon**	47.00	42.84	38.67	46.39
**Oxygenated compounds**	50.88	55.44	59.46	52.33
**Sesquiterpenes**	0.36	0.58	0.59	0.6

As shown in the tables, the concentration of oxygenated and sesquiterpenes compounds increased in MAHD method. Beside the concentration of monoterpene hydrocarbons decreased in comparison to HD. Ferhat *et al* ^27^, Lucchesi *et al* ^28^, Okoh *et al* ^29^ and Bendahou *et al* ^30^ showed that the content of oxygenated compounds in the oil obtained by Microwave Distillation (MD) was higher than in the oil obtained by HD. But Wang *et al* ^31^ reported that the content of oxygenated compounds of the oil obtained by MD was lower than that by HD for *Cuminum cyminum L*., and *Zanthoxylum bungeanum Maxim*. Therefore, the possible reason for this contradictory fact is that the content of oxygenated compounds of the oil was dependent on the species instead of the extraction method. During the procedure of MD, microwave irradiation highly accelerated the extraction process without causing considerable changes in the essential oil composition, although the percentages of some components depended on the technique applied.

Concentration of oxygenated compounds and monoterpene hydrocarbons increased by 17% and decreased by 17.7% with the increasing of 50% of microwave power comparing to HD method. Besides, the concentrations of these compounds decreased by 5.8% and increased by 8.3% in the presence of salt in MAHD method, respectively.

Because of aroma, flavor and therapeutic properties, oxygenated compounds can be used as measurements of essential oil quality. Monoterpene hydrocarbons are less valuable than oxygenated compounds in terms of their contribution to the fragrance of the essential oil ^31^. So, the higher amount of oxygenated compounds in MAHD indicates the higher quality of essential oil.

### Cost, cleanliness and safety considerations

The reduced cost of extraction is clearly advantageous for microwave method in terms of time and energy. The time needed for the complete extraction of rosemary essential oil was found as 30 *min* for MAHD and 90 *min* for HD. The extraction time was reduced by about 67% by using microwave. The reason for the reduction in the processing time is the heat generated by microwave heating which results in high pressure gradient inside the product. Microwaves interact selectively with the free water molecules present in the gland and vascular systems which leads to localized heating ^32^ . As a result of internal superheating, a dramatic expansion and consequently rupture of cell walls occurs allowing the extraction of essential oil.

The energy requirements to perform the extraction, based on maximum power consumption of electro mantle for HD and microwave oven for MAHD considering the total period of a full recovery, were 1500 *W* for HD and 180 *W* for MAHD. This indicates a substantial saving in the extraction cost when using MAHD instead of HD.

The calculated quantity of carbon dioxide released into the atmosphere is higher in the case of HD (1200 *g* CO _2 _) than for MAHD (144 *g* CO _2 _). These calculations were made according to the literature: to obtain 1 *kWh* from coal or other fossil fuel, 800 *g* CO _2_ will be released into the atmosphere during combustion ^27^ .

Therefore, MAHD is proposed as an “environmentally friendly” extraction method suitable for essential oil extraction.

## Conclusion

A similar extraction yield was achieved at significantly shorter extraction time when using MAHD instead of HD. In addition, MAHD method offers important advantages over traditional alternatives, namely shorter extraction times, substantial savings of energy and a reduced environmental hazard (less CO _2_ ejected in the atmosphere). The GC results showed that the amount of oxygenated compounds and monoterpene hydrocarbons increased and decreased, respectively by MAHD method. Also, increasing 50% microwave input power increased and decreased the concentration of oxygenated compounds and monoterpene hydrocarbons. This result leads to the fact that the quality of essential oil was increased by MAHD. So, MAHD can be considered as a green technology. MAHD is a good alternative for the extraction of essential oils from rosemary since it provides essential oils of similar quality compared to conventional HD while reducing the time and cost of the process drastically.
